# Comparative Effectiveness of Novel Combination Therapies for Simultaneous Management of Hypertension and Hypercholesterolemia: A Systematic Review and Meta-Analysis

**DOI:** 10.7759/cureus.71876

**Published:** 2024-10-19

**Authors:** Apurva Popat, Sweta Yadav

**Affiliations:** 1 Internal Medicine, Marshfield Clinic Health System, Marshfield, USA

**Keywords:** angiotensin receptor blockers, beta blockers, calcium channel blockers, combination therapy, hypercholesterolemia, hypertension, primary care, statins

## Abstract

Hypertension and hypercholesterolemia are the two most common modifiable risk factors for cardiovascular disease (CVD). Current guidelines recommend treating these risk factors simultaneously rather than in isolation. One prominent way to simultaneously treat the risk factors is by concurrently administering blood pressure (BP) lowering and lipid-lowering drugs (statins). However, there is still a controversy on which antihypertensive drugs to combine with statins for effective treatment. Therefore, the present meta-analysis assessed the efficacy of various antihypertensive agents combined with statins on BP and low-density lipoprotein cholesterol (LDL-C).

PubMed, MEDLINE, Cochrane Central Register of Controlled Trials (CENTRAL), and Google Scholar databases were searched thoroughly for records published in English up to February 2024. According to the PICOS (Patients, Intervention, Comparison, Outcomes, and Study design) criteria, randomized controlled trials (RCTs) evaluating the effectiveness of combination therapy of antihypertensives and statins in treating hypertension and hypercholesterolemia were eligible for inclusion. Furthermore, statistical analyses were performed using Review Manager software (RevMan version 5.4.1), and quality assessment was performed using the Cochrane risk of bias tool.

Eight RCTs comprising 1,182 patients with hypertension and hypercholesterolemia were included. Compared to statin monotherapy, no significant difference in the change of LDL-C levels was observed in patients receiving combination therapy of angiotensin receptor blockers (ARBs) with statins (MD, -3.98; *P* = 0.56) and BBs with statins (mean difference [MD], -0.47; *P* = 0.90). However, calcium channel blockers (CCBs) combined with statins showed a significantly greater reduction in LDL-C levels than statins alone (MD, -8.0; *P* = 0.0008). Similarly, patients treated with CCBs and statins had a considerable decrease in diastolic blood pressure (DBP) than those treated with antihypertensives only (MD, -6.37; *P *= 0.04). On the other hand, patients receiving antihypertensive drugs only demonstrated significantly better reductions in systolic blood pressure (SBP) than patients treated with combination therapy of ARBs and statins (MD, 2.88; *P *< 0.00001). Furthermore, we found that triple combination therapy was associated with better BP-lowering effects than double combination therapy of antihypertensive and statin (MD, -15.15; *P *< 0.00001 for SBP; and MD, -10.28; *P* < 0.00001, for DBP). No significant difference was recorded in the incidence of treatment-emergent adverse events.

Concurrent administration of antihypertensives and statins has similar effects on BP and LDL-C as the use of either drug alone. Furthermore, triple combination therapy (two antihypertensives and a statin) is associated with better BP-lowering effects than double combination therapy (one antihypertensive and a statin).

## Introduction and background

Hypertension and hypercholesterolemia are the two most common modifiable risk factors for cardiovascular disease (CVD). Research has shown that these conditions occur concurrently in up to 30% of patients with CVD, and their combined effect on mortality due to cardiovascular events is more significant than each condition alone [1-3]. Historically, managing hypertension and hypercholesterolemia involved separate pharmacological agents targeting each condition separately. However, current guidelines recommend that these risk factors be treated simultaneously rather than in isolation [4].

One prominent way of simultaneously treating hypertension and hypercholesterolemia is by administering a combination of blood pressure (BP)-lowering drugs (i.e., angiotensin-converting enzyme inhibitors, angiotensin II receptor blockers, beta-blockers [BBs], and calcium channel blockers) with lipid-lowering agents (statins). Statins represent the mainstay treatment for hypercholesterolemia as they are efficient in lowering low-density lipoprotein cholesterol (LDL-C) levels by inhibiting the 3-hydroxy-3-methylglutaryl-coenzyme A reductase (HMGR). Statins also present significant pleiotropic effects, such as ameliorating endothelial function, reducing oxidative injury, and modulating innate immunity [5]. Furthermore, statins can lower BP through upregulation of nitric oxide expression, decreased release of endothelin-1, and improved vascular stiffness [6]. This is evident in previous meta-analyses where statins have been linked with a significant reduction in BP, especially for patients presenting uncontrolled hypertension [7,8].

While evidence has shown that combining antihypertensives with lipid-lowering treatment is beneficial for hypertensive patients with hypercholesterolemia, there is still a controversy on which antihypertensives to combine with statins. Therefore, the present meta-analysis was designed to accumulate the available literature and compare the effectiveness of various antihypertensive agents combined with statins. Furthermore, the comparative effectiveness of triple combination therapies was assessed.

## Review

Methodology

Information Sources and Searches

PubMed, MEDLINE, Cochrane Central Register of Controlled Trials (CENTRAL), and Google Scholar databases were searched extensively for potential articles published from inception until February 2024. Additionally, reference lists of articles related to our topic were scoured for more studies. The search criteria used to identify studies from the electronic databases were as follows: (Hypertension OR high blood pressure) AND (hypercholesteremia OR hyperlipidemia) AND (antihypertensives OR blood pressure lowering drugs OR angiotensin-converting enzymes OR angiotensin II receptor blockers OR calcium channel blockers OR beta-blockers) AND (lipid-lowering drugs OR Statins). Furthermore, we eliminated all grey literature and duplicate articles as they would have interfered with the scientific purpose of the current study and undermined the statistical analyses.

Eligibility Criteria

Two independent reviewers assessed the full texts of retrieved articles and included those that met the following PICOS criteria (Population, Intervention, Comparison, Outcome, and Study Design).

Population: patients with concomitant hypertension and hypercholesterolemia

Intervention: any hypertensive drug combined with any lipid-lowering drug

Comparison: statins or antihypertensives only

Outcome: changes in BP (systolic blood pressure [SBP] and diastolic blood pressure [DBP] and changes in lipid profile [i.e., LDL-C])

Study design: randomized controlled trials (RCTs)

On the other hand, articles published in languages other than English, those designed as case reports, observational studies, reviews, editorials or conference abstracts, studies without comparative groups, and those, including patients with hypertension or hypercholesterolemia only were excluded. Additionally, studies that included patients with hypertension and dyslipidemia were excluded. Although in some cases, dyslipidemia and hypercholesterolemia are used interchangeably, hypercholesterolemia refers to having higher than normal levels of LDL-C or total cholesterol without including triglycerides, while dyslipidemia encompasses high levels of triglycerides, low levels of HDL-cholesterol, and qualitative lipid abnormalities. For this reason, we decided to eliminate studies on dyslipidemia.

Data Extraction

Two independent reviewers reviewed articles that met the inclusion criteria outlined earlier and extracted the data required for review and analysis in separate Excel files. Afterward, the data was harmonized and compiled in a single table. In the event of discrepancies in the extracted data, the reviewers engaged in constructive debates, and if they could not reach an agreement, a third reviewer was consulted. The data extracted included Author ID (surname of the primary author and the year of publication), study design, pertinent characteristics of included patients (sample size, age, and sex ratio), country in which the study was carried out, intervention, duration for outcome assessment, and evaluated outcomes.

Quality Appraisal

The current systematic review and meta-analysis included only RCTs; therefore, quality assessment was performed with the Cochrane Risk-of-Bias tool (RoB) embedded within the Review Manager software. For each RCT, the following domains were assessed: selection, attrition, performance, reporting, and other biases. Under these domains, a low risk of bias was assigned to a criterion fully addressed, while a high and unclear risk of bias was assigned to criteria not addressed entirely and those with insufficient information to make a clear judgment. Furthermore, the overall study quality was assessed by converting RoB scores to the Agency for Healthcare Research and Quality (AHRQ) standards [9].

Data Synthesis

All statistical analyses in this study were performed with the Review Manager software (RevMan version 5.4.1). A random effect model was fitted to all analyses to counter the anticipated heterogeneity and provide conservative effect sizes. The effect measure used for continuous data was the mean difference (MD), while the effect measure for dichotomous data was the simple odds ratio (OR). Moreover, a 95% confidence interval (CI) was employed in all statistical analyses, and statistical significance was represented by a *P*-value of less than 5% (*P* < 0.05). The interstudy heterogeneity was calculated using the *I*² statistic, with values above 50% regarded as substantial. Subgroup analyses were conducted whenever possible based on the class of antihypertensive agents used or the comparative interventions. When the change in blood pressure and lipid profiles was not reported directly, online calculators were used to estimate the changes.

Results

Study Selection

**Figure 1:**
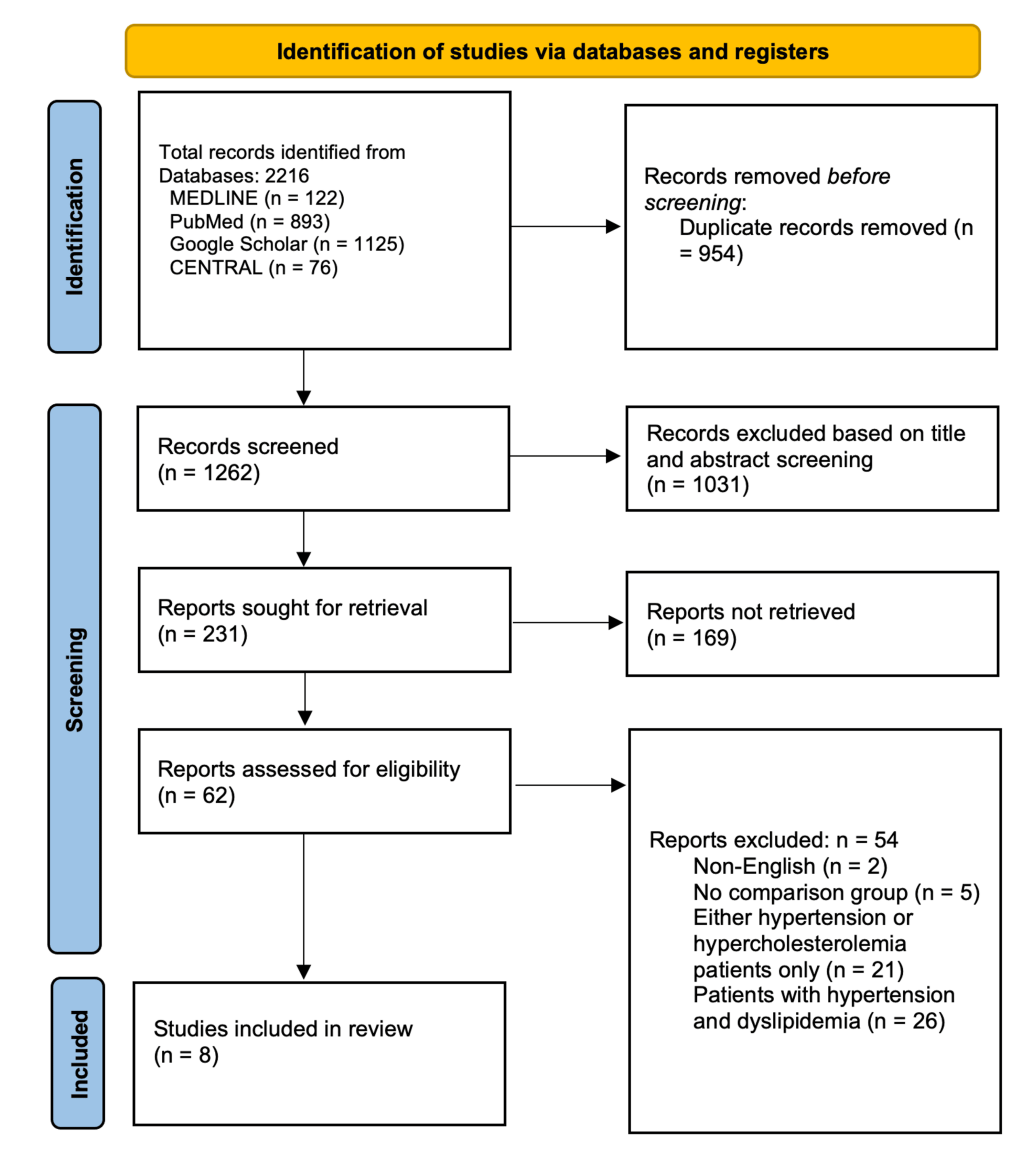
PRISMA flowchart for study selection. PRISMA, Preferred Reporting Items for Systematic Reviews and Meta-Analyses

After the initial database search, 2,216 records with the predefined MeSH terms were identified. An in-depth evaluation of these records led to the elimination of 954 articles deemed as close or exact duplicates. Out of the remaining 1,262 unique records, title and abstract screening led to the exclusion of a further 1,031 articles considered irrelevant to our study. Moreover, we did not retrieve 169 records because there were ongoing clinical trials, case reports, editorials, review articles, or conference abstracts. Finally, only 8 articles were eligible for review and analysis, while the other 54 were excluded due to the following reasons: 2 were published in different languages, 5 did not have comparison groups, 21 included patients with hypercholesterolemia or hypertension only, and 26 included patients with hypertension and dyslipidemia. This process of study selection is schematically depicted in full in the PRISMA flow diagram (Figure 1).

Summary of Study Characteristics

Eight RCTs, including 1,182 patients with hypertension and hypercholesterolemia, were used for review and analysis. Of the 8 RCTs, 6 focused on the comparative effectiveness of a double combination therapy (i.e., an antihypertensive combined with a statin), while 2 focused on triple combination therapy (i.e., two antihypertensives combined with a statin). Regarding the type of statin used, rosuvastatin was administered in 5 studies, atorvastatin in 2 studies, and simvastatin in 1 study. On the other hand, angiotensin receptor blockers (ARBs) were evaluated in 5 studies, calcium channel blockers (CCBs) in 4 studies, and BBs in one study.

**Table 1:** Summary of study characteristics. *Overall study quality was evaluated by converting risk-of-bias assessment scores to the Agency for Healthcare Research and Quality (AHRQ) standards [9]. NR, not reported; RCT, randomized controlled trial; DBP, diastolic blood pressure; SBP, systolic blood pressure; LDL-C, low-density lipoprotein cholesterol; TAEs, treatment-emergent adverse events

Author ID	Study design	Country	Patient characteristics			Intervention	Duration of outcome assessment (weeks)	Outcomes	Overall study quality*
			Sample (*n*)	M/F	Age (years)				
Cho et al. (2019) [10]	Multicenter, double-blind, parallel phase III RCT	Korea	212	125/87	NR	Fixed-dose combination therapy of candesartan and Rosuvastatin	8	Changes in DBP, SBP, and LDL cholesterol	Fair
Rhee et al. (2020) [11]	Multicenter, double-blind, parallel phase III RCT	Korea	376	209/167	62.25 ± 9.56	Combination therapy of Nebivolol and Rosuvastatin	8	Changes in DBP, SBP, and LDL cholesterol, and TAEs	Fair
Fogari et al. (2004) [12]	RCT	Italy	45	22/23	NR	Combination therapy of amlodipine and atorvastatin	12	Changes in DBP, SBP, and LDL cholesterol	Poor
Ge et al. (2008) [13]	RCT	China	126	52/74	NR	Combination therapy of amlodipine and atorvastatin	16	Changes in DBP, SBP, and LDL cholesterol	Poor
Han et al. (2007) [14]	Double-blind, placebo-controlled cross-over RCT	Korea	47	20/27	57 ± 2	Combination therapy of simvastatin and losartan	8	Changes in DBP, SBP, and LDL cholesterol	Poor
Jang et al. (2015) [15]	Multicenter, double-masked, double-dummy phase III RCT	Korea	101	85/16	61.5	Combination therapy of valsartan and rosuvastatin	8	Changes in DBP, SBP, and LDL cholesterol, and TAEs	Fair
Lee et al. (2017) [16]	Multicenter, double-blind, placebo-controlled RCT	Korea	143	107/36	59.85 ± 8.34	Triple combination therapy of amlodipine, losartan, and rosuvastatin	8	Changes in DBP, SBP, and LDL cholesterol, and TAEs	Fair
Kim et al. (2019) [17]	Multicenter, double-blind phase III RCT	Korea	132	93/39	65.6 ± 10.3	Triple combination therapy of telmisartan, amlodipine, and rosuvastatin.	8	Changes in DBP, SBP, and LDL cholesterol, and TAEs	Fair

Quality Assessment Outcomes

**Figure 2:**
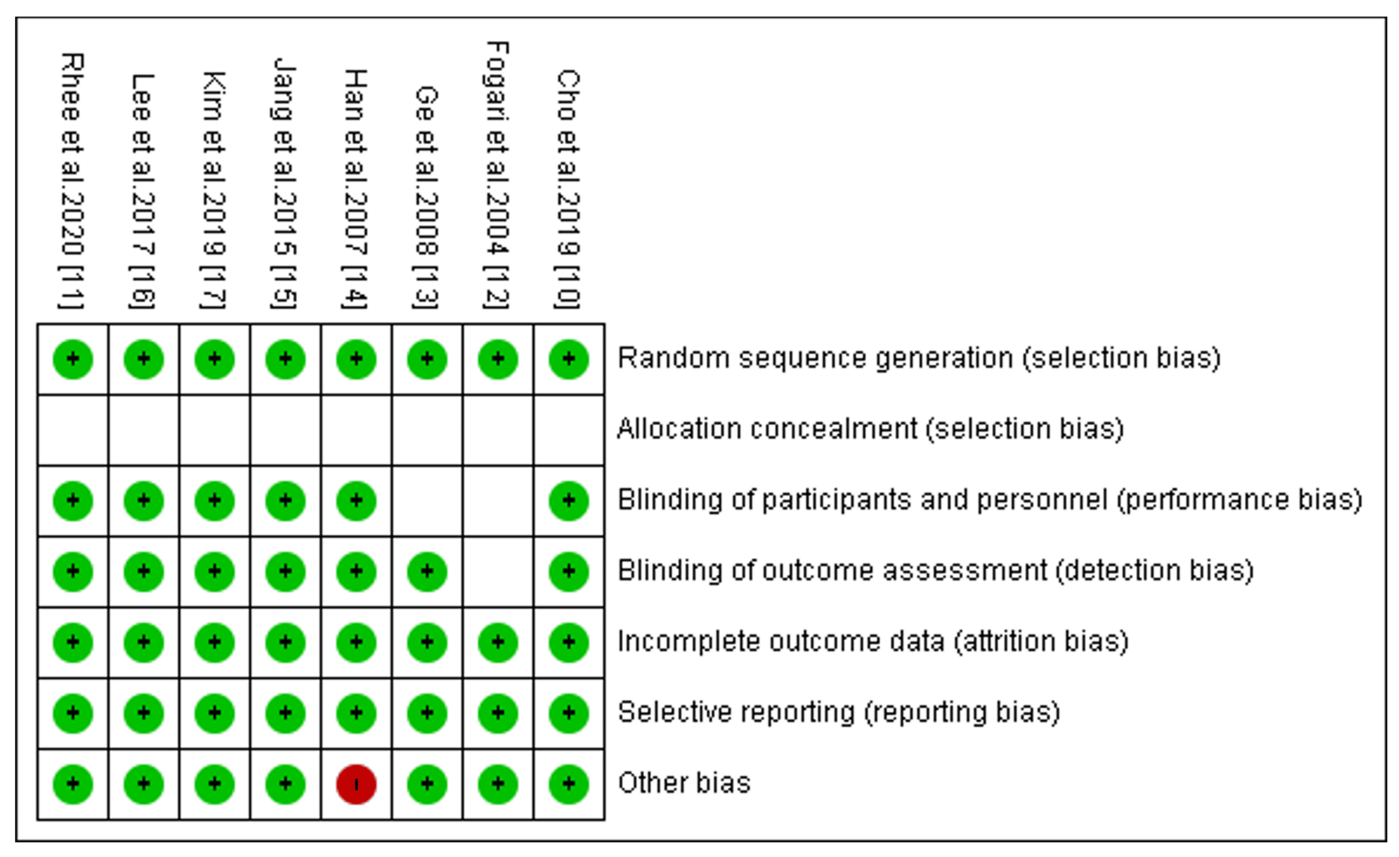
Risk-of-bias summary using the Cochrane Risk-of-Bias tool. The green color indicates low risk of bias, the red color indicates a high risk of bias, and no color was assigned for unclear risk of bias [9].

A summary of the risk-of-bias assessment is shown in Figure 2. From the assessment, we observed that all the trials had an unclear risk for selection bias because the studies did not provide sufficient information on the allocation of patients to make a clear judgment. Moreover, one clinical trial had a high risk of other bias because it employed a cross-over design that can influence the outcomes of our meta-analysis.

Comparative Efficacy of Novel Double Combination Therapies

**Figure 3:**
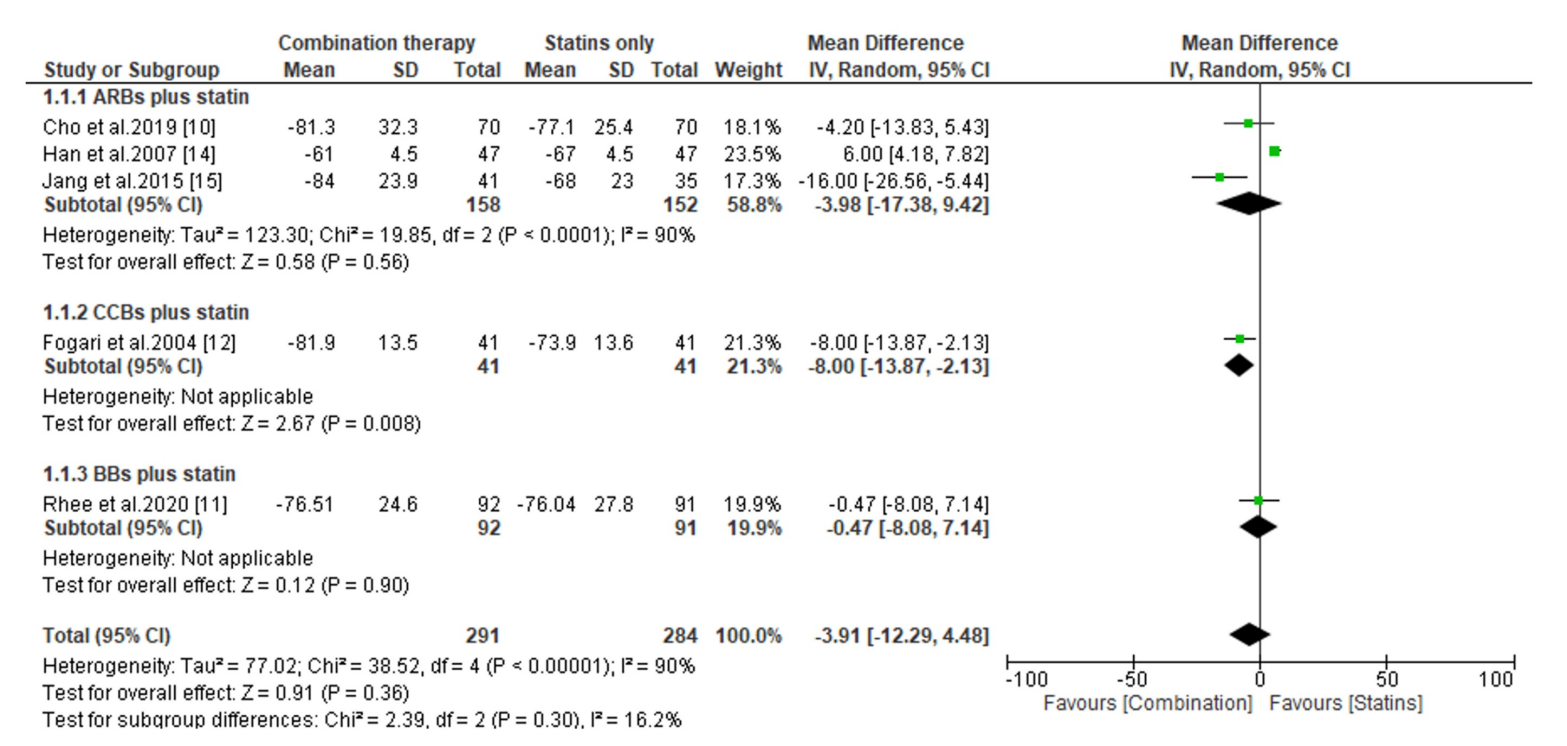
Change in LDL-C levels between combination therapies versus statin only. LDL-C, low-density lipoprotein cholesterol; CI, confidence interval; ARB, angiotensin receptor blocker; CCB, calcium channel blocker; BB, beta-blocker

**Figure 4:**
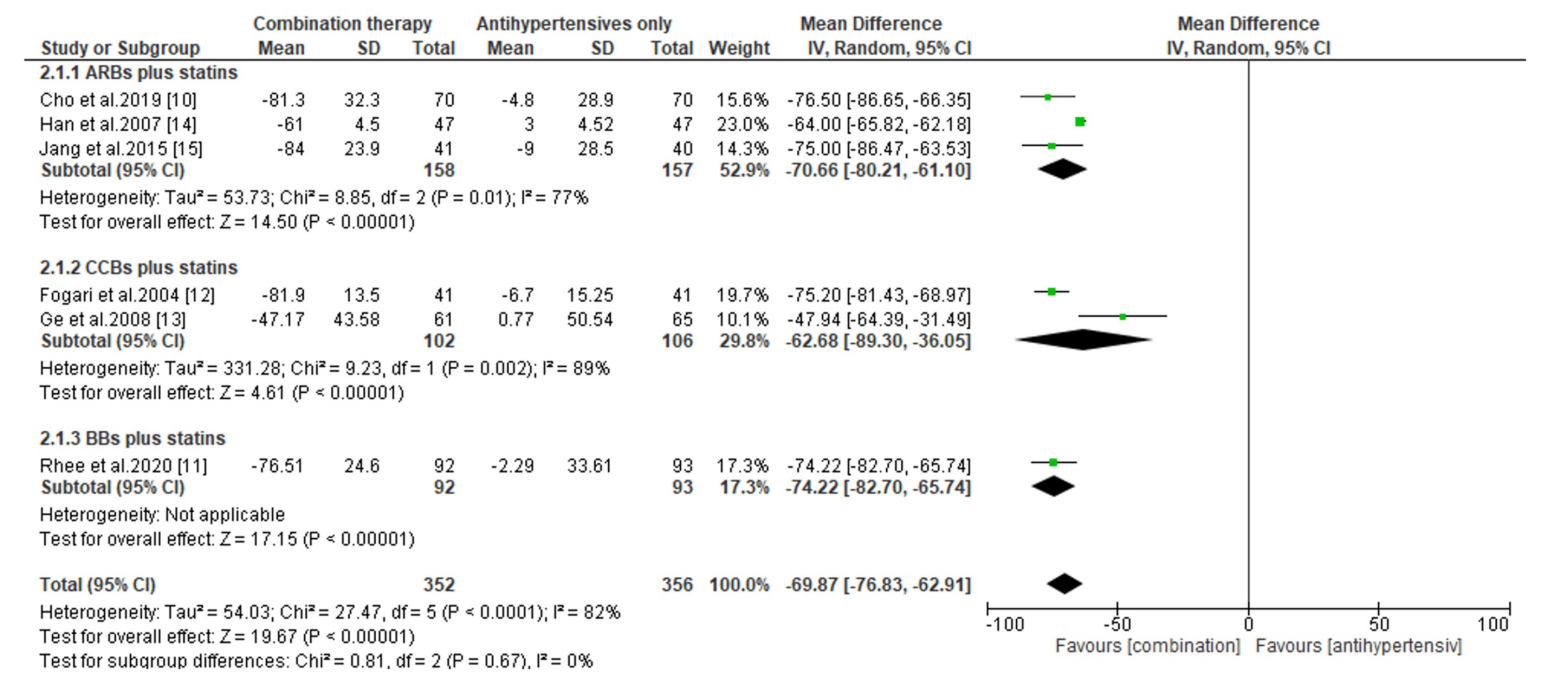
Change in LDL-C levels between combination therapies versus antihypertensives only. LDL-C, low-density lipoprotein cholesterol; CI, confidence interval; ARB, angiotensin receptor blocker; CCB, calcium channel blocker; BB, beta-blocker

**Figure 5:**
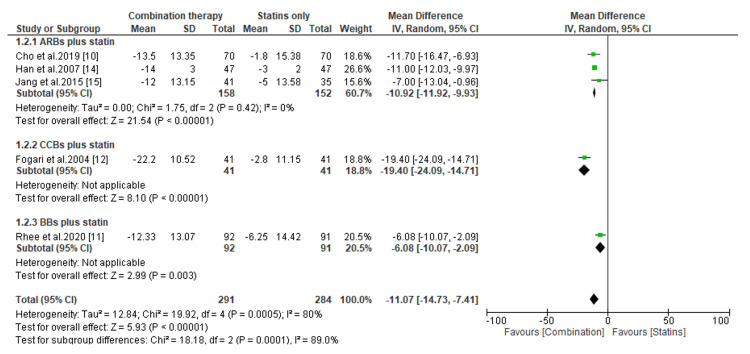
Change in SBP levels between combination therapies versus statin only. SBP, systolic blood pressure; CI, confidence interval; ARB, angiotensin receptor blocker; CCB, calcium channel blocker; BB, beta-blocker

**Figure 6:**
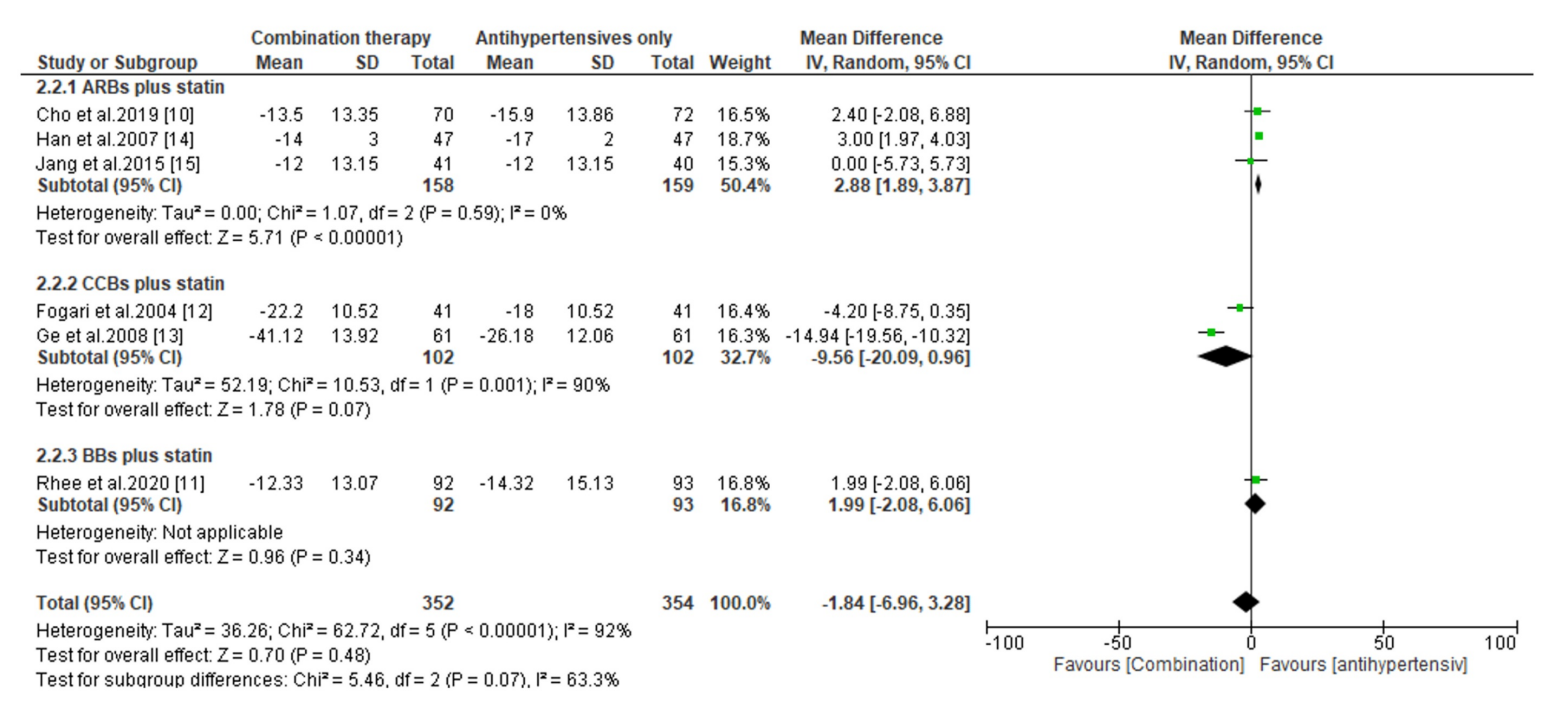
Change in SBP levels between combination therapies versus antihypertensives. SBP, systolic blood pressure; CI, confidence interval; ARB, angiotensin receptor blocker; CCB, calcium channel blocker; BB, beta-blocker

**Figure 7:**
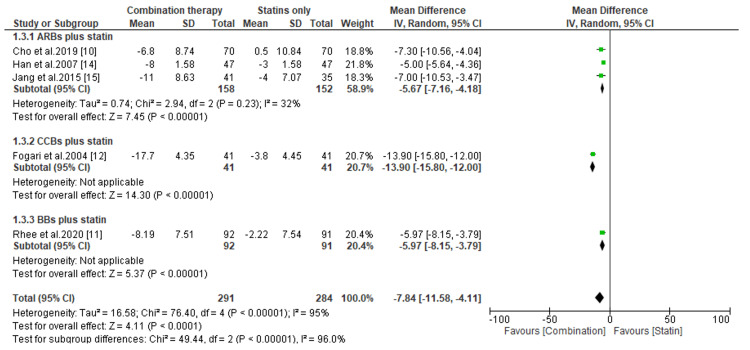
Change in DBP levels between combination therapies versus statin only. DBP, diastolic blood pressure; CI, confidence interval; ARB, angiotensin receptor blocker; CCB, calcium channel blocker; BB, beta-blocker

**Figure 8:**
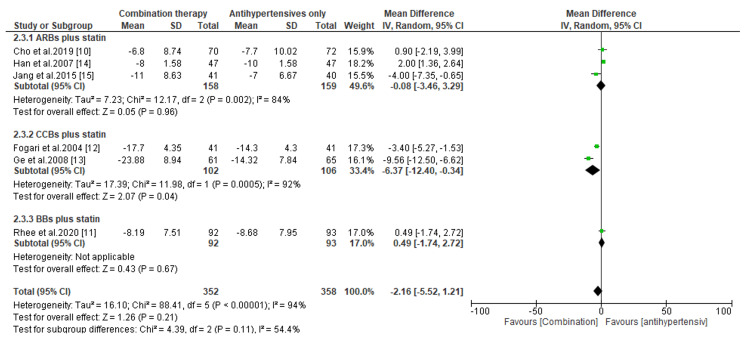
Change in DBP levels between combination therapies versus antihypertensives only. DBP, diastolic blood pressure; CI, confidence interval; ARB, angiotensin receptor blocker; CCB, calcium channel blocker; BB, beta-blocker

The comparative efficacy of double combination therapies on the reduction of LDL-C, SBP, and DBP are presented in Figures 3-8. Regarding the change in LDL-C levels, our subgroup analysis showed that patients receiving CCBs combined with statins have significantly better reduction in LDL-C levels than those treated with statins only (MD, -8.0; *P* = 0.0008) (Figure 3). However, compared to patients receiving statins only, the change in LDL-C was statistically insignificant for those treated with ARBs combined with statins (MD, -3.98; *P* = 0.56) and BBs combined with statins (MD, -0.47; *P* = 0.90) (Figure 3). On the other hand, we found that regardless of the type of antihypertensive administered, patients receiving combination therapies had significant reductions in LDL-C levels than those receiving antihypertensives only (*P* < 0.00001) (Figure 4).

The present meta-analysis has also shown that antihypertensive drugs combined with statins result in a considerable reduction in SBP compared to statins alone (MD, -10.92; *P *< 0.00001, for ARBs plus statins; MD, -19.40; *P *< 0.00001, for CCBs plus statins; and MD, -6.08; *P* =0.003, for BBs plus statins) (Figure 5). On the other hand, the subgroup analysis demonstrated an insignificant difference in SBP reduction for patients receiving CCBs plus statins and BBs plus statins compared to those receiving antihypertensives only (MD, -9.56; *P *= 0.07 and MD, 1.99; *P *= 0.34, respectively) (Figure 6). However, the pooled data suggested that patients receiving antihypertensives only tend to have significant reductions in SBP than patients treated with ARBs combined with statins (MD, 2.88; *P *< 0.00001) (Figure 6).

Regarding the change in DBP levels, we noticed a significant reduction in DBP levels among patients treated with combination therapy than those treated with statins only (MD, -5.67; *P *< 0.00001; MD, -13.90; *P *< 0.00001; and MD, -5.97; *P *< 0.00001, for ARBs plus statins, CCBs plus statins, and BBs plus statins, respectively) (Figure 7). Similarly, patients treated with CCBs and statins had a considerable reduction in DBP than those treated with antihypertensives only (MD, -6.37; *P *= 0.04) (Figure 8). However, compared to patients receiving antihypertensives only, the difference in DBP reduction was statistically insignificant among patients receiving ARBs plus statins and BBs plus statins (MD, -0.08; *P *= 0.96 and MD, 0.49; *P *= 0.67, respectively) (Figure 8).

Comparative Efficacy of Novel Triple Combination Therapies

**Figure 9:**
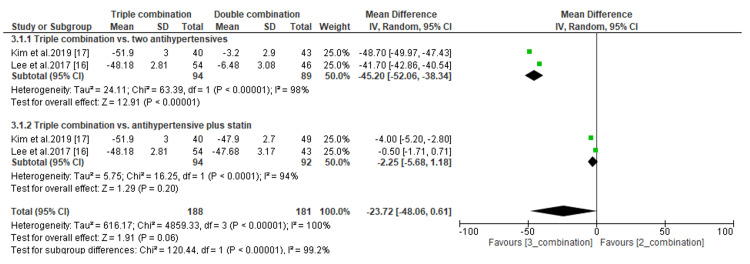
Comparison of percentage change in LDL-C between triple combination and double combination therapies. LDL-C, low-density lipoprotein cholesterol; CI, confidence interval

**Figure 10:**
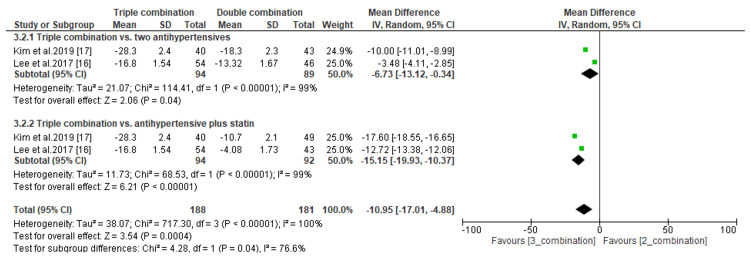
Comparison of least mean change in SBP between triple combination and double combination therapies. SBP, systolic blood pressure; CI, confidence interval

**Figure 11:**
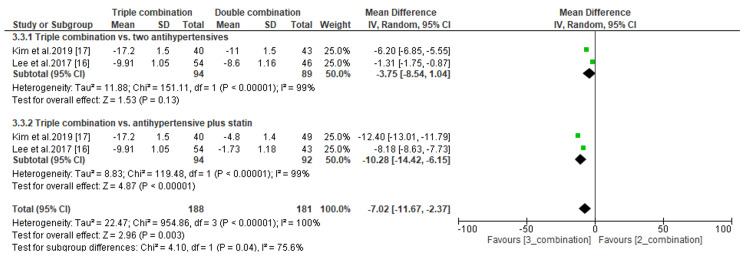
Comparison of least mean change in DBP between triple combination and double combination therapies. DBP, diastolic blood pressure; CI, confidence interval

The efficacy of triple combination therapies was assessed by comparing the least mean percentage change in LDL-C and the least mean change in SBP and DBP levels. Regarding the percentage change in LDL-C, we found that the difference was significant when compared to antihypertensive drugs only (MD, -45.50; *P *< 0.00001) but not compared to antihypertensives plus statins (MD, -2.25; *P *= 0.20) (Figure 9). In addition, the subgroup analysis demonstrated a significant difference in the least mean change in SBP between patients receiving triple combination therapies and those receiving either two antihypertensives only (MD, -6.73; *P *= 0.04) or an antihypertensive plus a statin (MD, -15.15; *P *< 0.00001) (Figure 10). Similarly, the difference in the least mean change in DBP was statistically significant between patients receiving triple combination therapies and those receiving an antihypertensive and a statin (MD, -10.28; *P *< 0.00001) (Figure 11). However, the difference was not significant compared to patients receiving antihypertensive drugs only (MD, -3.75; *P *= 0.13) (Figure 11).

Tolerability of Novel Combination Therapies

**Figure 12:**
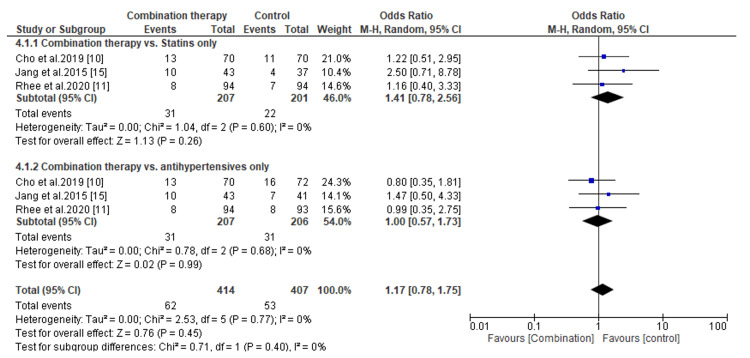
Comparison of incidences of treatment-emergent adverse events between combination therapy and antihypertensive or statin alone. CI, confidence interval

**Figure 13:**
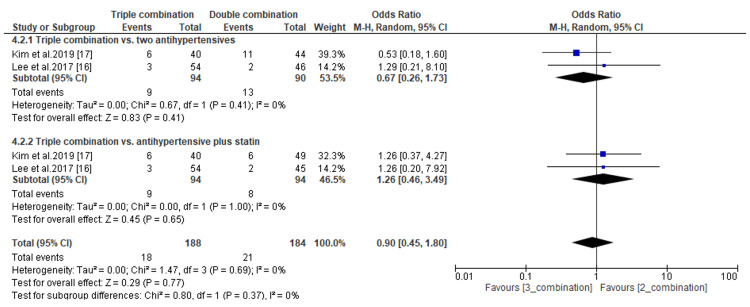
Comparison of incidences of treatment-emergent adverse events between triple combination therapy and double combination therapy. CI, confidence interval

The tolerability of combination therapies was assessed by evaluating the incidences of treatment-emergent adverse events (TEAs). The pooled analysis of data from three included studies showed no significant difference in TEAs between patients receiving antihypertensives combined with statins and those treated with statins or antihypertensives only (OR, 1.41; *P *= 0.26; and OR, 1.0; *P *= 0.99, respectively) (Figure 12). Similarly, the incidence of TEAs did not differ statistically between patients receiving triple combination therapies and those receiving double combination therapies of antihypertensives only or antihypertensives plus statins (OR, 0.67; *P *= 0.41; and OR, 1.26; *P *= 0.65, respectively) (Figure 13).

Discussion

Hypertension and hypercholesterolemia are well known to induce endothelial dysfunction, which is a pre-clinical marker for cardiovascular disease [18]. Therefore, simultaneous management of these conditions is an excellent step towards minimizing major cardiovascular events. In the present meta-analysis, we investigated whether the co-administration of antihypertensive drugs and statins is beneficial for patients presenting with hypertension and hypercholesterolemia.

Comparative Effectiveness of Double Combination Therapies

This study has demonstrated that when antihypertensive drugs and statins are administered concurrently in hypertensive hypercholesteremic patients, the effect on LDL-C does not differ significantly compared to statins alone. This finding aligns with what was reported in previous meta-analyses [19,20]. However, further analysis showed that CCBs, specifically amlodipine, combined with statins (atorvastatin) resulted in a significantly higher reduction in LDL-C than statin monotherapy. This finding was also observed by Messerli et al., who noted that among patients with hypertension and dyslipidemia, coadministration of atorvastatin and amlodipine was associated with greater reductions in LDL-C and total cholesterol than atorvastatin alone [21]. The exact reason for this improvement in lipid profile among patients receiving amlodipine and atorvastatin is still unknown, and further clinical trials are required to explore this finding. However, it can be associated with the fact that the atorvastatin and amlodipine combination produces a synergistic reduction in oxidative damage to human LDL, an effect that is not always observed in other combinations of antihypertensives and statins [22].

Regarding BP control, administering antihypertensive drugs concurrently with statins does not result in a significant reduction in SBP compared to antihypertensives only. This finding is also collaborated by previous meta-analyses [19,20]. However, our subgroup analysis showed that antihypertensive drugs alone result in a greater reduction in SBP than a combination of ARBs and stains. This finding was heavily weighted in by the study carried out to investigate the effects of simvastatin combined with losartan [14]. However, there is still a need for more RCTs to explore the exact reason for this outcome, as previous studies evaluating the combination of simvastatin and losartan have found contradicting information. For example, an experimental study found that after 8 weeks of treatment, hypertensive hypercholesterolemic rats receiving combined therapy (simvastatin and losartan) had significantly greater reductions in SBP than those treated with losartan only [23].

Interestingly, we noticed that patients treated with CCBs combined with statins have significantly greater reductions in DBP than those receiving antihypertensive drugs alone. This finding can be attributed to the significant reduction in DBP among patients receiving amlodipine-atorvastatin combination in the study by Ge and colleagues, which shows that the combination therapy might have presented several specific synergistic vascular effects. First, the concurrent administration of amlodipine and atorvastatin in vitro has been shown to stimulate the release of nitric oxide from the endothelial cells, resulting in a decrease in BP [24]. Secondly, in hypertensive patients, atorvastatin can help lower BP independently of its cholesterol-lowering effect. This blood-pressure-lowering effect can be attributed to the fact that statins have been shown to improve endothelium-dependent vascular function and cause considerable vasodilation, which leads to a significant increase in the sensitivity of the vessel wall to the vasodilating action of amlodipine. Finally, atorvastatin was seen to decrease levels of serum high-sensitivity C-reactive protein (hs-CRP) and uric acid, and the combination of atorvastatin and amlodipine also decreased other inflammatory markers [25], which might have resulted in reduced blood pressure.

Comparative Effectiveness of Triple Combination Therapies

The present meta-analysis has also shown that triple combination therapies have significant BP-lowering effects than double combination therapy of an antihypertensive drug and a statin. Although the exact reason for this difference is unknown, one of the studies used for the analysis investigated factors such as sex, age, baseline BP and lipid levels, concurrent medications, and underlying comorbidities and found none of these factors could explain the difference [17]. The only explanation for the significant difference in BP reduction among the triple combination group is the administration of rosuvastatin. While there is no evidence from large-scale and well-designed RCTs to support the BP-lowering effect of rosuvastatin, some studies have reported a pleiotropic effect of statins in hypertensive patients with hypercholesterolemia [26-29]. A previous meta-analysis of RCTs only reported that statins cause a considerable reduction in SBP, and the BP-lowering effect is even greater when the baseline BP is high [30]. Similarly, Briasoulis et al. conducted a meta-analysis evaluating data from 40 prospective RCTs and found small but statistically significant reductions in SBP among patients taking statins [8]. The mechanism and the extent of BP reduction with the administration of statins is not elucidated, but it is believed that statins reduce BP by increasing the bioavailability of nitric oxide and improving arterial compliance [8].

In terms of improvements in lipid profile, the current meta-analysis has shown that triple combination therapy has a similar effect on LDL-C as the double combination therapy (antihypertensive drug plus statin). Therefore, these results, combined with the BP-lowering effect, suggest that adding two antihypertensive drugs to a statin therapy does not attenuate the lipid-lowering effect of the statin. Furthermore, triple combination therapy does not affect tolerability as the incidence of TAEs did not differ compared to the antihypertensive-statin combination. Although the triple combination therapy seems to improve BP in hypertensive patients with hypercholesterolemia, replicating these positive effects in routine practice is rather very difficult due to lack of treatment adherence. This is evident in clinical trials where as low as one-third of the patients persist in taking their medications 6 months after the initiation of antihypertensive and lipid-lowering therapy [31]. The best way to improve medication adherence is by administering fixed combinations from the onset or switching as soon as possible during treatment. In this regard, the UMPIRE trial found that a fixed-dose combination therapy of aspirin, statin, and antihypertensive agents resulted in significantly higher adherence to medication and improvement in serum LDL-C and SBP [32]. Similarly, two phase III clinical trials evaluating the fixed-dose combination of irbesartan/atorvastatin [33] and rosuvastatin/valsartan [15] reported high compliance to treatment among patients with hypertension and hypercholesterolemia.

Limitations

The outcomes presented in this meta-analysis should be interpreted cautiously due to several limitations. First, most statistical analyses in the current study had high interstudy heterogeneity. This heterogeneity can be attributed to variations in sample sizes, different drug regimens, and differences in administered dosages. However, we used the random effects model to counter this heterogeneity and provide more conservative effect sizes. Second, only articles published in English were used for review and analysis, meaning that our study was subject to reporting bias because relevant data published in other languages was omitted. Third, the follow-up duration of the included trials was relatively short (i.e., ranging from 8 to 16 weeks); therefore, the long-term effects of combination therapies remain unclear. Fourth, we did not carry out a meta-analysis on complex outcomes such as cardiovascular and cerebrovascular events due to underreporting of these outcomes in the included studies. Therefore, future research articles should assess the potential effects of combining antihypertensive drugs with statins on cardiovascular and cerebrovascular events. Finally, we also included crossover trials in our analysis; therefore, it is possible that the effects of one treatment may have been carried over to the other treatment and influenced our meta-analytic results.

## Conclusions

In summary, concurrent administration of antihypertensives and statins has similar effects on BP and LDL-C as the use of antihypertensives and statins alone. However, evidence suggests that CCBs, specifically amlodipine, when administered concurrently with statins (atorvastatin), can lead to a greater reduction in LDL-C than statin alone. Furthermore, it seems that triple combination therapy (two antihypertensives and a statin) is associated with better BP-lowering effects than double combination therapy (one antihypertensive and a statin). However, further research is required to establish this finding and evaluate the BP-lowering effects of rosuvastatin in triple combination therapy.
